# Proportion of early extubation and short-term outcomes after esophagectomy: a retrospective cohort study

**DOI:** 10.1097/JS9.0000000000000568

**Published:** 2023-06-21

**Authors:** Yuki Hirano, Takaaki Konishi, Hidehiro Kaneko, Hidetaka Itoh, Satoru Matsuda, Hirofumi Kawakubo, Kazuaki Uda, Hiroki Matsui, Kiyohide Fushimi, Hiroyuki Daiko, Osamu Itano, Hideo Yasunaga, Yuko Kitagawa

**Affiliations:** a Department of Hepatobiliary–Pancreatic and Gastrointestinal Surgery, International University of Health and Welfare School of Medicine, Chiba; b Department of Clinical Epidemiology and Health Economics, School of Public Health; c Department of Cardiovascular Medicine, The University of Tokyo, Bunkyo-ku; d Department of Surgery, Keio University School of Medicine, Shinjyuku-ku; e Department of Health Policy and Informatics, Tokyo Medical and Dental University Graduate School, Bunkyo-ku; f Division of Esophageal Surgery, National Cancer Center Hospital, Chuo-ku, Tokyo, Japan

**Keywords:** early extubation, esophageal cancer, oesophagectomy, respiratory complications

## Abstract

**Background:**

The proportion of early extubation after esophagectomy varies among hospitals; however, the impact on clinical outcomes is unclear. The aim of this retrospective study was to evaluate associations between the proportion of early extubation in hospitals and short-term outcomes after esophagectomy. Because there is no consensus regarding the optimal timing for extubation, the authors considered that hospitals’ early extubation proportion reflects the hospital-level extubation strategy.

**Materials and methods:**

Data of patients who underwent oncologic esophagectomy (July 2010–March 2019) were extracted from a Japanese nationwide inpatient database. The proportion of patients who underwent early extubation (extubation on the day of surgery) at each hospital was assessed and grouped by quartiles: very low- (<11%), low- (11–37%), medium- (38–83%), and high-proportion (≥84%) hospitals. The primary outcome was respiratory complications; secondary outcomes included reintubation, anastomotic leakage, other major complications, and hospitalization costs. Multivariable regression analyses were performed, adjusting for patient demographics, cancer treatments, and hospital characteristics. A restricted cubic spline analysis was also performed for the primary outcome.

**Results:**

Among 37 983 eligible patients across 545 hospitals, early extubation was performed in 17 931 (47%) patients. Early extubation proportions ranged from 0–100% across hospitals. Respiratory complications occurred in 10 270 patients (27%). Multivariable regression analyses showed that high- and medium-proportion hospitals were significantly associated with decreased respiratory complications [odds ratio, 0.46 (95% CI, 0.36–0.58) and 0.43 (0.31–0.60), respectively], reintubation, and hospitalization costs when compared with very low-proportion hospitals. The risk of anastomotic leakage and other major complications did not differ among groups. The restricted cubic spline analysis demonstrated a significant inverse dose-dependent association between the early extubation proportion and the risk of respiratory complications.

**Conclusion:**

A higher proportion of early extubation in a hospital was associated with a lower occurrence of respiratory complications, highlighting a potential benefit of early extubation after esophagectomy.

## Introduction

Highlights

- Among 37 893 esophagectomies across 545 hospitals, early extubation was performed in 47% of patients.
- Early extubation proportions ranged from 0–100% across hospitals.
- Hospitals with high and medium proportions of early extubation were associated with decreased respiratory complications, reintubation, and hospitalization costs, compared to those with very low proportions.
- These results suggest the potential benefits of the early extubation strategy after esophagectomy.

Esophagectomy is the mainstay of treatment for resectable esophageal cancer; however, the procedure has a high-risk of complications, especially respiratory complications^1^. Although the incidence of respiratory complications has recently decreased with the widespread use of minimally invasive esophagectomy and enhanced recovery after surgery (ERAS) programs, they remain the most common complications after esophagectomy^2^. Preventing respiratory complications is particularly important, as previous studies have demonstrated their association with poor overall survival, as well as postoperative mortality after esophagectomy^3,4^.

Extubation after prophylactic overnight mechanical ventilation following esophagectomy was historically standard practice based on the assumption that this would minimize the risk of respiratory complications^5,6^. However, early extubation has become the preferred strategy in some hospitals as it can safely improve short-term outcomes when compared with conventional extubation, even in highly invasive surgeries. A previous single-center cohort study (*n*=96) showed that compared with conventional extubation strategy, early extubation strategy after esophagectomy did not increase the risk of reintubation and respiratory complications; additionally, it was associated with decreased catecholamine use and length of ICU stay, and increased early mobilization^7^. A recent meta-analysis (*n*=566) including five single-center retrospective studies also showed that early extubation did not increase the risk of reintubation, complications, or length of hospital stay; however, the evidence level was considered low to very low (using the GRADE system^8^)^9^. Randomized controlled trials and large-scale retrospective studies comparing early and conventional extubation are lacking; thus, the safety and effectiveness of early extubation strategy remain uncertain, and there is no consensus regarding the optimal timing for extubation after esophagectomy. Additionally, although recent ERAS programs for esophagectomy address intraoperative management under ventilation, there are no direct recommendations for early extubation^10^.

In reality, the use of early or conventional extubation after esophagectomy may vary widely among hospitals^7^. Some hospitals with routine early extubation strategy achieve early extubation in more than 90% of patients, while other hospitals have either never performed early extubation (instead using the conventional extubation strategy), or perform it selectively (selective early extubation strategy)^7,11–13^. We thus considered that the proportion of early extubation in a hospital reflects the hospital-level extubation strategy. In the present study, we aimed to examine the association between the proportions of early extubation performed at each hospital, and short-term outcomes after oncological esophagectomy using a nationwide inpatient database.

## Materials and methods

### Data source

This retrospective cohort study was conducted using the Diagnosis Procedure Combination database, a nationwide inpatient database in Japan^14^. This database collects more than eight million discharge abstracts and hospital administrative claims data annually from over 1200 healthcare facilities. All university hospitals are required to participate in the database, whereas other hospitals can participate voluntarily. The requirement for informed consent was waived because of the anonymous nature of the data. This study was approved by the institutional review board with a unique identifying number of the registration.

The database includes the following information: sex; age; body mass index (BMI); smoking index; diagnosis and comorbidities on admission and complications after admission, recorded using the International Classification of Diseases, Tenth Revision (ICD-10) codes; clinical cancer stage based on the seventh edition of the Union for International Cancer Control Tumor, Node, Metastasis classification; use of preoperative chemotherapy/radiotherapy; medication during hospitalization; interventional/surgical procedures according to the original Japanese codes; unique hospital identifier; and discharge status. The discharge abstracts for each patient were coded by the attending physicians. Previous validation studies have shown high accuracy for patients with esophageal cancer^15^, surgical procedures including esophageal cancer surgery^16^, comorbidities^17^, and postoperative complications^18^.

### Study protocol

Patients who underwent esophagectomy with two-field (thoraco-abdominal) or three-field (cervico-thoraco-abdominal) lymph node dissection for esophageal cancer between July 2010 and March 2019 were identified from the database using the original Japanese procedure codes. Patients who underwent transhiatal esophagectomy and two-stage reconstruction were not included in this study. The exclusion criteria were as follows: age less than 18 years, combined surgery for laryngeal or hypopharyngeal cancer, missing data on BMI, having received general anesthesia on the day after esophagectomy (i.e. received general anesthesia for esophagectomy overnight, or for other surgical procedures on the day after esophagectomy), and discharge on the day of or the day after esophagectomy (to avoid immortal bias). We also excluded patients who underwent surgery at hospitals that performed less than nine esophagectomies during the study period (July 2010–March 2019). We did not exclude patients with stage IV cancer, as esophageal cancer with supraclavicular lymph node metastasis is considered curable in Japan^19^; however, such metastases are classified as stage IV with distant metastases in the Union for International Cancer Control classification.

We examined the proportion of patients who underwent early extubation at each hospital during the study period^20–22^; early extubation was defined as extubation on the day of surgery without requiring mechanical ventilation on the day after surgery. Hospitals were categorized into four groups with approximately equal numbers of patients in each group according to the proportions of early extubation: very low (<11%); low (11–37%); medium (38–83%); high (≥84%).

The primary outcome was respiratory complications, identified using relevant ICD-10 codes (J12–18, J80, J96, J690, J691, J958, J959) or mechanical ventilation use lasting greater than 2 days following surgery^20^. Secondary outcomes included the use of mechanical ventilation for greater than 2 days and greater than 7 days (defined as mechanical ventilation use lasting >2 days and >7 days following surgery, respectively), reintubation (defined as unplanned postoperative intubation), the use of noninvasive ventilation and high-flow nasal cannula, anastomotic leakage, other major complications (defined as pneumothorax, chylothorax, empyema, peritonitis, ileus, bowel obstruction, symptomatic hiatal or diaphragmatic hernia, pulmonary embolism, acute coronary syndrome, heart failure, stroke, acute kidney injury, sepsis, and others resulting in death)^23,24^, in-hospital mortality, vasopressor use (defined as continuous use of noradrenaline, dopamine, or dobutamine on the day of and the day after surgery)^21^, postoperative length of stay, and total hospitalization costs. The ICD-10 and procedure codes used to define these postoperative complications are shown in Supplemental Table 1, Supplemental Digital Content 3, http://links.lww.com/JS9/A728. Information on high-flow nasal cannula was not available until April 2016, when it was reimbursed. In the analyses of high-flow nasal cannula use, patients who underwent esophagectomy before April 2016 were excluded.

We investigated patient demographics, cancer treatments, and hospital characteristics (Supplemental Appendix 1, Supplemental Digital Content 3, http://links.lww.com/JS9/A728). The hospital volume was defined as the number of esophagectomies performed per year in each hospital, and was categorized into quartiles.

### Statistical analysis

First, we conducted multivariable regression analyses fitted with generalized estimating equations, including logistic regression analyses for binary outcomes, and linear regression analyses for continuous outcomes. The generalized estimating equations enabled us to adjust the analyses for clustering of variables within the same hospital, such as patient background characteristics and physician practice patterns^25^. The explanatory variables in the multivariable analyses were patient demographics (sex, age, BMI, smoking index, Barthel index, Charlson comorbidity index, diabetes mellitus, hypertension, chronic obstructive pulmonary disease, renal failure, liver disease, corticosteroids use before surgery, and clinical cancer stage), cancer treatments (preoperative chemotherapy, preoperative radiotherapy, field of esophagectomy, thoracic approach, reconstruction organ, vessel reconstruction, epidural anesthesia, prophylactic corticosteroids, and duration of anesthesia), and hospital characteristics (hospital type, hospital volume, and fiscal year). Odds ratios (ORs) and their 95% CIs were calculated in the logistic regression analyses, and beta coefficients and their 95% CIs were calculated in the linear regression analyses for each hospitals’ early extubation proportion category (reference group: very low-proportion).

Second, we performed subgroup analyses using multivariable logistic regression analyses fitted with generalized estimating equations stratified by sex (male/female), age (<70 or ≥70 years), BMI (<18.5 or≥18.5 kg/m^2^), smoking index (0–20 or≥21 pack/years), Charlson comorbidity index (2 or≥3), clinical stage (0–I/II–IV), preoperative chemotherapy (no/yes), preoperative radiotherapy (no/yes), field of lymph node dissection (two-field/three-field), thoracic approach (open or minimally invasive), epidural anesthesia (no/yes), prophylactic corticosteroids (no/yes), duration of anesthesia (<585 or≥585 min), and hospital volume (<7.3 or≥7.3 cases per year) for the primary outcome. For each subgroup analysis, the proportion of hospitals’ early extubation was assessed and grouped into quartiles.

Third, we performed a restricted cubic spline (RCS) analysis to visualize the relationship between the hospital proportion (as a continuous variable) and the primary outcome. A linear association between the exposure and the outcome cannot be assumed a priori, and we assumed a nonlinear association between them. While ordinary regression analysis using hospitals’ early extubation proportion categories may lose information and power, RCS analysis without categorization of the proportion allows for using all data points to estimate the dose-response association between the proportion and the outcomes^26,27^. We used the 5th, 35th, 65th, and 95th percentiles of hospitals’ early extubation proportion as the four knots for the cubic spline^28^; the explanatory variables were the same as those in the main analyses. We also fitted generalized estimating equations to the RCS analysis and calculated the OR and 95% CI for each hospitals’ early extubation proportion relative to the reference proportion of 0%.

In the summary statistics, the Kruskal–Wallis *H* test was used to compare continuous variables, and the *χ* ^2^-test was used for comparisons of categorical variables between groups. The Cochran–Armitage trend test was used to evaluate the change in the proportion of patients undergoing early extubation over the study period. Statistical significance was accepted at *P*<0.05, and all statistical analyses were conducted using STATA version 17 (StataCorp LLC, College Station). This study was conducted in line with the strengthening the reporting of cohort, cross-sectional and case-control studies in surgery (STROCSS) criteria^29^, Supplemental Digital Content 1, http://links.lww.com/JS9/A726.

## Results

Overall, 40 245 patients were identified; 2262 patients who met the following exclusion criteria were excluded: age less than 18 years (*n*=1), combined surgery for laryngeal/hypopharyngeal cancer (*n*=466), missing data on BMI (*n*=344), received general anesthesia on the day after esophagectomy (*n*=193), discharge on the day of and the day after surgery (*n*=9), and underwent surgery at hospitals with greater than nine esophagectomies during the study period (*n*=1249). As a result, 37 983 patients remained for analyses. Among 545 hospitals, early extubation was performed in 17 931 patients (47%). Supplemental Figure 1, Supplemental Digital Content 2, http://links.lww.com/JS9/A727 shows a trend toward an increase in the proportion of early extubation over time (43 in 2010 to 53% in 2018; *P*<0.001). The proportion of patients undergoing early extubation ranged from 0–100% across hospitals. Table 1 shows baseline characteristics (patient demographics, treatments, and hospital characteristics) according to the quartile of hospitals’ early extubation proportion. Hospitals with high proportions of early extubation were more likely to use epidural anesthesia and have shorter durations of anesthesia, as well as be high-volume and non-teaching hospitals.

**Table 1:** Baseline characteristics and crude outcomes categorized by hospitals’ early extubation proportion.

Variables	Very low <11% 122 hospitals (*n*=9407)	Low 11–37% 161 hospitals (*n*=9395)	Medium 38–83% 164 hospitals (*n*=9621)	High ≥84% 98 hospitals (*n*=9560)	*P*
*Patient demographics*					
Male sex	7820 (83)	7835 (83)	8052 (84)	7916 (83)	0.40
Age, year					
18–59	1656 (18)	1767 (19)	1747 (18)	1825 (19)	0.092
60–64	1647 (18)	1654 (18)	1693 (18)	1654 (17)	
65–69	2311 (25)	2139 (23)	2334 (24)	2205 (23)	
70–74	2063 (22)	2063 (22)	2080 (22)	2057 (22)	
≥75	1730 (18)	1772 (19)	1767 (18)	1819 (19)	
BMI, kg/m^2^					
<16.0	372 (4.0)	356 (3.8)	354 (3.7)	341 (3.6)	0.018
16.0–18.4	1504 (16)	1475 (16)	1402 (15)	1359 (14)	
18.5–22.9	4813 (51)	4841 (52)	5018 (52)	4998 (52)	
23.0–27.4	2388 (25)	2414 (26)	2482 (26)	2541 (27)	
≥27.5	330 (3.5)	309 (3.3)	365 (3.8)	321 (3.4)	
Smoking index, pack-years					
0–5	2658 (28)	2763 (29)	2807 (29)	2608 (27)	<0.001
6–20	1018 (11)	991 (11)	1035 (11)	1244 (13)	
21–40	2132 (23)	2049 (22)	2144 (22)	2352 (25)	
≥41	2554 (27)	2270 (24)	2434 (25)	2657 (28)	
Missing	1045 (11)	1322 (14)	1201 (12)	699 (7.3)	
Barthel index <95	668 (7.1)	232 (2.5)	236 (2.5)	201 (2.1)	<0.001
Charlson comorbidity index					
2	7226 (77)	7175 (76)	7537 (78)	7883 (82)	<0.001
3–4	1328 (14)	1477 (16)	1508 (16)	1023 (11)	
≥5	853 (9.1)	743 (7.9)	576 (6.0)	654 (6.8)	
Hypertension	1954 (21)	2153 (23)	2120 (22)	2409 (25)	<0.001
Diabetes mellitus	1216 (13)	1264 (13)	1327 (14)	1020 (11)	<0.001
COPD	174 (1.9)	284 (3.0)	243 (2.5)	136 (1.4)	<0.001
Renal failure	91 (1.0)	99 (1.1)	86 (0.9)	65 (0.7)	0.042
Liver disease	471 (5.0)	524 (5.6)	517 (5.4)	389 (4.1)	<0.001
Corticosteroid use before surgery	78 (0.8)	86 (0.9)	82 (0.9)	48 (0.5)	0.005
Clinical stage					
0–I	2895 (31)	2861 (30)	2979 (31)	2785 (29)	<0.001
II–IV	6406 (68)	6065 (65)	6385 (66)	6657 (70)	
X or missing	106 (1.1)	469 (5.0)	257 (2.7)	118 (1.2)	
*Treatments*					
Preoperative treatment					
Chemotherapy	4683 (50)	4015 (43)	4270 (44)	4932 (52)	<0.001
Radiotherapy	751 (8.0)	542 (5.8)	642 (6.7)	748 (7.8)	<0.001
Three-field lymph node dissection	7806 (83)	7353 (78)	7675 (80)	7770 (81)	<0.001
Thoracic approach					
Unspecified (to March 2014)	3566 (38)	3944 (42)	3675 (38)	3443 (36)	<0.001
Open	2394 (29)	2564 (27)	2572 (27)	2670 (28)	
Minimally invasive	3147 (33)	2887 (31)	3374 (35)	3447 (36)	
Intestine reconstruction	230 (2.4)	229 (2.4)	263 (2.7)	226 (2.4)	0.37
Vessel reconstruction	43 (0.5)	25 (0.3)	21 (0.2)	10 (0.1)	<0.001
Epidural anesthesia	7450 (79)	7200 (77)	7778 (81)	8399 (88)	<0.001
Prophylactic corticosteroids	6853 (73)	5715 (61)	5255 (55)	5967 (62)	<0.001
Duration of anesthesia, min					
<488	1567 (17)	2130 (23)	2046 (21)	3713 (39)	<0.001
488–584	2371 (25)	2327 (25)	2319 (24)	2441 (26)	
585–689	2615 (28)	2470 (26)	2502 (26)	1947 (20)	
≥690	2854 (30)	2468 (26)	2754 (29)	1459 (15)	
*Hospital characteristics*					
Teaching hospital	5980 (64)	6067 (65)	6385 (66)	4750 (50)	<0.001
Hospital volume, cases/year					
<7.3	1652 (18)	2793 (30)	2773 (29)	1265 (13)	<0.001
7.3–19.7	2558 (27)	2748 (29)	2482 (26)	2092 (22)	
19.8–42.8	2716 (29)	2788 (30)	1796 (19)	2315 (24)	
≥42.9	2481 (26)	1066 (11)	2570 (27)	3888 (41)	
Fiscal year					
2010–2011	1211 (13)	1687 (18)	1453 (15)	1449 (15)	<0.001
2012–2013	2355 (25)	2257 (24)	2222 (23)	1994 (21)	
2014–2015	2505 (27)	2319 (25)	2430 (25)	2579 (27)	
2016–2018	3336 (35)	3132 (33)	3516 (37)	3538 (37)	
*Crude outcomes*					
Respiratory complications	3237 (34)	3050 (32)	2495 (26)	1488 (16)	<0.001
Mechanical ventilation >2 days	1924 (20)	1914 (20)	1264 (13)	647 (6.8)	<0.001
Mechanical ventilation >7 days	899 (9.6)	950 (10)	729 (7.6)	466 (4.9)	<0.001
Reintubation	908 (9.7)	1072 (11)	687 (7.1)	459 (4.8)	<0.001
Noninvasive ventilation	83 (0.9)	158 (1.7)	74 (0.8)	49 (0.5)	<0.001
High-flow nasal cannula (from April 2016)^*^	253/3336 (7.6)	153/3132 (4.9)	128/3516 (3.6)	97/3538 (2.7)	<0.001
Anastomotic leakage	1392 (15)	1623 (17)	1240 (13)	1176 (12)	<0.001
Other major complications	1018 (11)	1131 (12)	1033 (11)	850 (8.9)	<0.001
In-hospital mortality	248 (2.6)	281 (3.0)	221 (2.3)	204 (2.1)	0.001
Vasopressor use^†^	5256 (56)	5152 (55)	4185 (44)	3429 (36)	<0.001
Postoperative length of stay, days	27 (19–43)	26 (19–45)	26 (18–41)	21 (16–33)	<0.001
Total hospitalization costs, US$	31 412 (27 551–39 187)	31 415 (27 219–40 157)	29 826 (25 934–37 809)	27 464 (24 506–32 789)	<0.001

Data are presented as *n* (%) or median (interquartile range).

* Patients who underwent esophagectomy before April 2016 (*n*=24 461) were excluded from the outcome analysis.

† Vasopressor use was defined as the use of noradrenaline, dopamine, or dobutamine on the day of, or the day after surgery.

COPD, chronic obstructive pulmonary disease.

Table 1 also shows the crude primary and secondary outcomes, while Supplemental Table 2, Supplemental Digital Content 3, http://links.lww.com/JS9/A728 shows the detailed postoperative complications. Overall, respiratory complications, reintubation, and in-hospital mortality occurred in 10 270 patients (27%), 3126 patients (8.2%), and 954 patients (2.5%), respectively. The percentages of patients with respiratory complications were 34 (3237/9407), 32 (3050/9395), 26 (2495/9621), and 16% (1488/9560) in very low-, low-, medium-, and high-proportion hospitals, respectively. The proportions of respiratory complications, mechanical ventilation for greater than 2 days and greater than 7 days, reintubation, noninvasive ventilation use, high-flow nasal cannula use, anastomotic leakage, other major complications, in-hospital mortality, vasopressor use, postoperative length of stay, and total hospitalization costs differed significantly among the groups.

Multivariable regression analyses demonstrated that hospitals with high and medium proportions of early extubation were significantly associated with a lower proportion of respiratory complications [OR, 0.46 (95% CI, 0.36–0.58) and 0.43 (0.31–0.60), respectively], compared to those with very low proportions (Table 2). Hospitals with high and medium proportions of early extubation were also associated with lower proportions of mechanical ventilation for greater than 2 days [OR, 0.35 (0.27–0.45) and 0.53 (0.42–0.68), respectively], and greater than 7 days [OR, 0.63 (0.51–0.79) and 0.75 (0.62–0.91), respectively], reintubation [OR, 0.68 (0.49–0.93) and 0.46 (0.22–0.94), respectively], and vasopressor use [OR, 0.52 (0.41–0.67) and 0.62 (0.47–0.81), respectively]; a shorter postoperative length of stay; and lower total hospitalization costs. The adjusted risk of noninvasive ventilation use, high-flow nasal cannula use, anastomotic leakage, other major complications, and in-hospital mortality did not differ significantly among the groups.

**Table 2:** Multivariable regression analyses for short-term outcomes.

	Low (11–37%)		Medium (38–83%)		High (≥84%)	
Variables	OR or β (95% CI)	*P*	OR or β (95% CI)	*P*	OR or β (95% CI)	*P*
Respiratory complications	0.93 (0.64–1.36)	0.72	0.43 (0.31–0.60)	<0.001	0.46 (0.36–0.58)	<0.001
Mechanical ventilation >2 days	0.81 (0.63–1.03)	0.088	0.53 (0.42–0.68)	<0.001	0.35 (0.27–0.45)	<0.001
Mechanical ventilation >7 days	0.94 (0.78–1.15)	0.57	0.75 (0.62–0.91)	0.003	0.63 (0.51–0.79)	<0.001
Reintubation	1.46 (0.76–2.81)	0.25	0.46 (0.22–0.94)	0.034	0.68 (0.49–0.93)	0.017
Noninvasive ventilation	2.05 (0.37–11.41)	0.41	1.06 (0.42–2.67)	0.91	0.49 (0.15–1.58)	0.23
High-flow nasal cannula (from April 2016)^*^	0.87 (0.50–1.51)	0.62	0.71 (0.39–1.32)	0.28	0.56 (0.26–1.20)	0.13
Anastomotic leakage	1.04 (0.88–1.22)	0.66	0.87 (0.73–1.03)	0.11	0.97 (0.82–1.15)	0.75
Other major complications	1.01 (0.88–1.15)	0.92	0.89 (0.77–1.02)	0.082	0.95 (0.82–1.10)	0.51
In-hospital mortality	0.96 (0.76–1.20)	0.70	0.80 (0.63–1.02)	0.075	1.06 (0.83–1.36)	0.63
Vasopressor use^†^	1.03 (0.81–1.31)	0.81	0.62 (0.47–0.81)	0.001	0.52 (0.41–0.67)	<0.001
Postoperative length of stay, d	−0.9 (−3.5 to 1.7)	0.50	−3.1 (−5.8 to −0.3)	0.028	−3.9 (−6.7 to −1.2)	0.005
Total hospitalization costs, US$	−400 (−1775 to 973)	0.57	−2052 (−3476 to −627)	0.005	−2823 (−4232 to −1415)	<0.001

OR and β are with reference to the very low (<11%) group.

* Patients who underwent esophagectomy before April 2016 (*n*=24 461) were excluded from the outcome analysis.

† Vasopressor use was defined as the use of noradrenaline, dopamine, or dobutamine on the day of or the day after surgery.

The explanatory variables were sex, age, BMI, smoking index, Barthel index, Charlson comorbidity index, diabetes mellitus, hypertension, chronic obstructive pulmonary disease, renal failure, liver disease, corticosteroids use before surgery, clinical cancer stage, preoperative chemotherapy, preoperative radiotherapy, field of esophagectomy, thoracic approach, reconstruction organ, vessel reconstruction, epidural anesthesia, prophylactic corticosteroids, duration of anesthesia, hospital type, hospital volume, and fiscal year. A generalized estimating equation was used to adjust for within-hospital clustering.

OR, odds ratio; β, beta coefficient.

**Figure 1:**
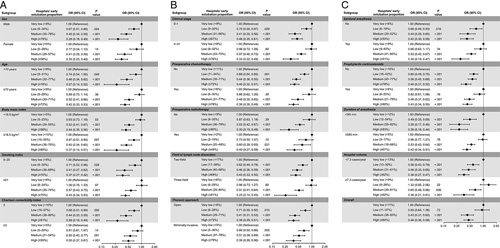
Subgroup multivariable regression analyses of respiratory complications (A–C). Odds ratios of respiratory complications associated with hospitals’ early extubation proportion. OR, odds ratio.

Figure 1 shows subgroup analyses for the primary outcome. All subgroup analysis-derived respiratory complications were in favor of hospitals with high and medium proportions of early extubation compared to those with very low proportions.

**Figure 2:**
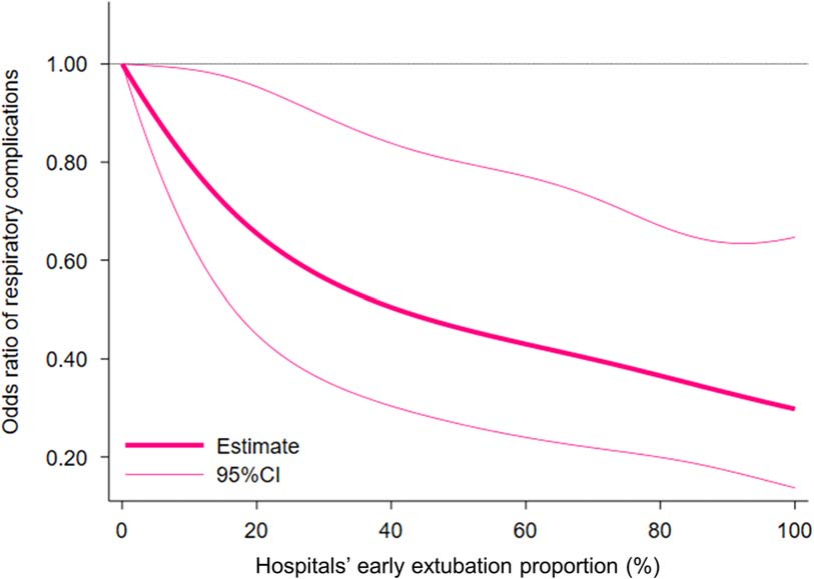
Adjusted dose-response association between hospitals’ early extubation proportion and respiratory complications in analyses using restricted cubic splines. Odds ratios for respiratory complications according to hospitals’ early extubation proportion relative to the reference proportion of 0%. Estimates are shown with 95% CIs.

Figure 2 illustrates the adjusted dose-response associations between hospitals’ early extubation proportion and the primary outcome in the RCS analysis, demonstrating a significant inverse dose-dependent association between the early extubation proportion and the risk of respiratory complications. Hospitals with an early extubation proportion of 11, 38, and 84% had a significantly lower risk of respiratory complications [OR, 0.78 (0.62–0.99); 0.51 (0.31–0.85); and 0.35 (0.19–0.65), respectively] than those with a proportion of 0%.

## Discussion

This study is the first to analyze the association between hospitals’ early extubation proportions and short-term outcomes after esophagectomy. Using a nationwide inpatient database, we investigated the associations between hospitals’ early extubation proportion and short-term outcomes among 37 983 esophagectomies for esophageal cancer, with adjustment for potential confounders (including hospital volume). Hospitals with high and medium proportions of early extubation were associated with decreased respiratory complications, use of mechanical ventilation for greater than 2 days and greater than 7 days, and reintubation; a shorter postoperative length of stay; and lower total hospitalization costs when compared with hospitals with very low proportions.

Prophylactic overnight ventilation after highly invasive surgeries—such as esophagectomy and cardiac surgery—was historically adopted in many hospitals^5,6,30^. Despite increasing interest in early extubation after esophagectomy, there is little evidence to support it. On the contrary, regarding cardiac surgery, previous randomized controlled trials and meta-analyses have shown that early extubation strategies reduce ICU stays, length of stay, and costs without increasing the risks of reintubation, complications, or mortality^30–33^. Accordingly, the ERAS programs for cardiac surgery recommend early extubation strategies^32,33^, which have been widely accepted in the last decade^34^. Thus, we consider that evidence on early extubation strategy following esophagectomy can modify the existing ERAS programs and improve the outcomes of patients with esophageal cancer.

In the current study, we investigated the association between hospitals’ early extubation proportion and short-term outcomes after oncologic esophagectomy. We considered that the proportion reflected the hospital-level extubation strategy; that is, physicians in a hospital with a high-proportion routinely conduct early extubation. The current analysis thus demonstrates that the early extubation strategy was associated with better respiratory outcomes (e.g. respiratory complications and prolonged mechanical ventilation) than the conventional extubation strategy. There are several possible mechanisms for this association; First, early extubation may have contributed to early postoperative mobilization^7^. The ERAS programs for esophagectomy strongly recommend early mobilization (as soon as possible) to prevent complications associated with bed rest, such as respiratory complications^10^. Second, ventilator-associated pneumonia may have decreased due to the short duration of mechanical ventilation^35^. Finally, early extubation may have reduced postoperative fluid infusions and prevented excess fluid by eliminating the need for overnight sedation; sedative (e.g. propofol)-induced cardiovascular depression generally requires increased fluid infusion^33^, resulting in pulmonary edema and respiratory complications^10,36^. Indeed, a lower occurrence of cardiovascular depression in hospitals with an early extubation strategy may have been attributed to less postoperative vasopressor use. Therefore, the early extubation strategy would be preferable in oncologic esophagectomy. Notably, the associations between hospitals’ early extubation proportion and respiratory complications were consistent in all subgroups, and the early extubation strategy may even be beneficial in patients at high-risk of respiratory complications (e.g. patients aged ≥70 years, received preoperative radiotherapy, undergoing open esophagectomy, and in low-volume hospitals)^37,38^. Furthermore, high and medium proportions of early extubation were also associated with decreased postoperative length of stay and total hospitalization costs. The early extubation strategy may also optimize hospital resource utilization.

Physicians at a hospital with a conventional extubation strategy may fear the risk of reintubation after early extubation. Previous studies showed that reintubation was associated with increased in-hospital mortality, length of stay, and hospitalization costs^39^. Moreover, postoperative reintubation was accompanied by technical difficulty and complications (i.e. hypoxia, hypotension, and esophageal intubation) when compared to the initial intubation for elective surgery in the same patient^40^. In particular, esophageal intubation after esophagectomy can cause critical damage to the anastomosis; however, the current study showed that hospitals with high and medium proportions of early extubation were associated with a decreased risk of postoperative reintubation, similar to the reduction in respiratory complications. This result suggests that the early extubation strategy is safe and may reduce, rather than increase, the risk of reintubation. Therefore, we recommend that hospitals with routine conventional extubation strategy implement an early extubation strategy. Still, conventional extubation may be beneficial in selected patients, such as those with bleeding requiring fluid resuscitation, or high-risk airways^41^.

Although high and medium proportions of early extubation were associated with a decreased risk of other outcomes—including respiratory complications—the risk of in-hospital mortality did not differ among groups. This may be because higher proportions of early extubation did not affect the risk of anastomotic leakage—the second most common complication associated with postoperative mortality^42^—or other major complications. Additionally, hospitals with very low proportions of early extubation may have relatively high experience in managing patients with respiratory complications, consequently avoiding failure to rescue following the complications owing to early detection and intensive care.

This study has several limitations; First, due to confounding, we did not perform direct comparisons between patients who underwent early and conventional extubation. In hospitals that use the early extubation strategy, patients who were not extubated early may have failed early extubation, or were at a high-risk due to intraoperative events. Instead, we focused on the difference in the proportions of early extubation among hospitals, and demonstrated the association between the proportions and short-term outcomes. Second, the proportions of early extubation may partially reflect hospitals’ performance regarding perioperative care. Hospitals with higher proportions may have been more diligent to reduce opioids doses, titrate anesthetic depth with bispectral index monitoring, avoid volume overload and hypothermia, and use multimodal analgesia. These perioperative care strategies would facilitate early extubation and concurrently directly reduce the risk of complications^10,33^. Indeed, hospitals with higher proportions were more likely to use epidural analgesia (an important component of multimodal analgesia). Moreover, we considered that hospitals’ early extubation proportion could be an important metric to assess perioperative care in esophagectomy, as in cardiac surgery^43–46^. Third, while the use of epidural analgesia was adjusted in the current analysis, we were unable to obtain information on paravertebral analgesia, which may be an option for multimodal analgesia after esophagectomy^47,48^. However, because paravertebral analgesia was uncommon in Japan during the study period^49^, the lack of information would not affect the results. Fourth, information on hospital factors other than teaching hospital status and hospital volume—such as the number of hospital beds, ICU availability, and nurse-patient ratio—was unavailable in the database. Reportedly, although the impact of these hospital factors on respiratory complications and reintubation remains unclear, these factors may be associated with death after major complications (i.e. failure to rescue) following major surgery, including esophagectomy^50^. Therefore, the additional hospital factors warrant inclusion in further studies. Fifth, information on preoperative lung function was unavailable in our database. However, lung function has been reported to be inversely related to pack-years of cigarette use (i.e. smoking index)^51,52^; thus, the current study was adjusted for smoking index. Sixth, information on the end time of surgery and the time interval in hours between esophagectomy and extubation was unavailable in our database. Thus, we used the calendar day to define early extubation and excluded patients who received general anesthesia for esophagectomy overnight. Moreover, because the end time of surgery may affect the extubation strategy, we adjusted for the duration of anesthesia in the current study. We consider that the duration of anesthesia would be a substitute explanatory variable for the end time of surgery since esophagectomy in Japan usually begins in the morning. Finally, because minimally invasive esophagectomy has been reimbursed separately from open esophagectomy by the publicly provided universal insurance system since April 2014, information on the thoracic approach was not included in the database for patients prior to April 2014. Compared to open esophagectomy, minimally invasive esophagectomy has been reported to reduce the risk of respiratory complications^53,54^. However, the associations between hospitals’ early extubation proportion and the primary outcome were consistent in the subgroup analysis when stratified by the specific thoracic approach.

## Conclusion

A higher proportion of early extubation in a hospital was associated with a lower occurrence of respiratory complications. Moreover, the higher proportion was also associated with fewer cases of prolonged ventilation use and reintubation, a shorter postoperative length of stay, and lower total hospitalization costs. These results suggest the potential benefits and safety of the early extubation strategy in patients undergoing esophagectomy for esophageal cancer.

## Ethical approval

This study was approved by the Institutional Review Board of the University of Tokyo [approval number: 3501-(3)].

## Consent

Written informed consent was obtained from the patient for publication of this case report and accompanying images. A copy of the written consent is available for review by the Editor-in-Chief of this journal on request.

## Sources of funding

This work was supported by grants from the Ministry of Health, Labor and Welfare, Japan (21AA2007 and 22AA2003) and the Ministry of Education, Culture, Sports, Science and Technology, Japan (20H03907).

## Author contribution

Y.H.: conceptualization, data curation, formal analysis, investigation, methodology, project administration, visualization, writing – original draft; T.K.: data curation, methodology, validation, writing – review and editing; H.K.: data curation, methodology, writing – review and editing; H.I.: data curation, validation, writing – review and editing; S.M. and H.K.: methodology, writing – review and editing; K.U., H.M., and K.F.: data curation, resources, writing – review and editing; H.D. and O.I.: supervision, writing – review and editing; H.Y.: conceptualization, funding acquisition, resources, supervision, writing – review and editing; Y.K.: conceptualization, supervision, writing – review and editing.

## Conflicts of interest disclosure

Dr Kitagawa has received grants and personal fees from Asahi Kasei Pharma Corporation; grants, personal fees, and others from Ono Pharmaceutical Co. Ltd.; grants and personal fees from Otsuka Pharmaceutical Factory Inc.; grants and personal fees from Nippon Covidien Inc.; grants, personal fees, and others from Taiho Pharmaceutical Co. Ltd.; grants, personal fees, and others from Chugai Pharmaceutical Co. Ltd.; grants and personal fees from Kaken Pharmaceutical Co. Ltd.; personal fees from AstraZeneca K.K.; personal fees from Ethicon Inc.; personal fees from Olympus Corporation; personal fees from Shionogi & Co. Ltd.; personal fees and others from Bristol-Myers Squibb K.K.; personal fees from MSD K.K.; personal fees from Smith & Nephew K.K.; personal fees from Aska Pharmaceutical Co. Ltd.; personal fees from Miyarisan Pharmaceutical Co. Ltd.; personal fees from Toray Industries, Inc.; personal fees from Daiichi Sankyo Co. Ltd.; personal fees from Chugai Foundation for Innovative Drug Discovery Science; personal fees from Nippon Kayaku Co. Ltd.; grants from Yakult Honsha Co. Ltd.; grants from Otsuka Pharmaceutical Co. Ltd.; grants from Tsumura & Co.; grants from Sumitomo Pharma Co. Ltd.; grants and personal fees from EA Pharma Co. Ltd.; grants from Eisai Co. Ltd.; grants from Kyouwa Hakkou Kirin Co. Ltd.; grants from Medicon Inc.; grants from Takeda Pharmaceutical Co. Ltd.; grants from Teijin Pharma Ltd.; and personal fees from Intuitive Surgical G.K., outside the submitted work. Dr. Konishi received grants from Pfizer Co. Ltd., Kanzawa Medical Research Foundation, and Japan Kampo Medicines Manufacturers Association outside the submitted work. There are no other conflicts of interest to disclose.

## Research registration unique identifying number (UIN)

1. Name of the registry: UMIN.
2. Unique Identifying number or registration ID: UMIN000050263.
3. Hyperlink to your specific registration (must be publicly accessible and will be checked): https://center6.umin.ac.jp/cgi-open-bin/ctr_e/ctr_view.cgi?recptno=R000057240.

## Guarantor

Yuki Hirano.

## Data availability statement

Because the data in this study was extracted from a nationwide database, data use requires approval of the Ministry of Health, Labor and Welfare, Japan.

## Provenance and peer review

Not commissioned, externally peer-reviewed.

## Supplementary Material

SUPPLEMENTARY MATERIAL
