# Supplementary figures and images for: Proportion of early extubation and short-term outcomes after esophagectomy: a retrospective cohort study
Source: Int J Surg. 2023 Jun 21;109(10):3097–106. doi: 10.1097/JS9.0000000000000568 (PMC10583926; doi:10.1097/JS9.0000000000000568)

## Slide 1
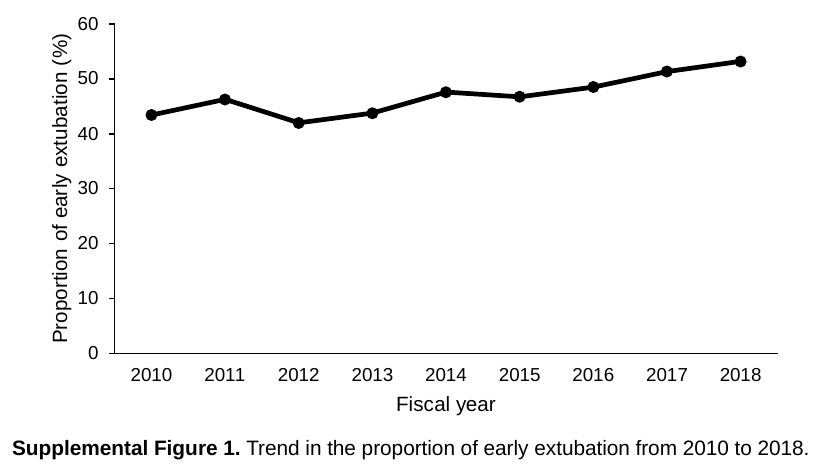

Supplemental Figure 1. Trend in the proportion of early extubation from 2010 to 2018.

Supplement: SUPPLEMENTARY MATERIAL [file js9-109-3097-s002.pptx]
