# Supplementary material for: Proportion of early extubation and short-term outcomes after esophagectomy: a retrospective cohort study
Source: Int J Surg. 2023 Jun 21;109(10):3097–106. doi: 10.1097/JS9.0000000000000568 (PMC10583926; doi:10.1097/JS9.0000000000000568)
Supplement: SUPPLEMENTARY MATERIAL [file js9-109-3097-s003.docx]

**Proportion of Early Extubation and Short-term Outcomes after Esophagectomy: A Retrospective Cohort Study**

**Supplemental Appendix 1.** Descriptions of background characteristics

**Supplemental Table 1.** ICD-10 and procedure codes used to identify postoperative complications

**Supplemental Table 2.** Subcategories of other major complications categorized by hospitals’ early extubation proportion

**Supplemental Appendix 1. Descriptions of background characteristics**

We examined patients’ background factors, including sex, age, body mass index classification, smoking index, activities of daily living (Barthel index <95), comorbidities (Charlson comorbidity index, diabetes mellitus, hypertension, diabetes mellitus, chronic obstructive pulmonary disease, renal failure, and liver disease), corticosteroid use before surgery, and clinical cancer stage. Age was categorized into five groups: 18–59, 60–64, 65–69, 70–74, and ≥75 years; and the smoking index was categorized into five groups: 0–5, 6–20, 21–40, ≥41 pack-years, and missing. Comorbidities were scored according to the Charlson comorbidity index using the protocol established by Quan et al. using ICD-10 codes,^1^ and classified into three groups: 2, 3–4, and ≥ 5. The Charlson comorbidity index is a method of predicting mortality by classifying and weighting comorbidities, and has been validated and is widely used in national studies worldwide.^2,3^ The clinical cancer stage was divided into three categories: 0–I, II–IV, and X/missing. Body mass index was categorized into five groups based on the criteria for Asia-Pacific populations by the World Health Organization according to our previous study: < 16.0 kg/m^2^ (severe underweight), 16.0–18.4 kg/m^2^ (mild-to-moderate underweight), 18.5–22.9 kg/m^2^ (normal), 23.0–27.4 kg/m^2^ (overweight), and ≥ 27.5 kg/m^2^ (obese).^4,5^

We also investigated characteristics of cancer treatment, such as preoperative treatment (chemotherapy/radiotherapy), field of lymph node dissection, surgical approach, reconstruction used (stomach/intestine), vessel reconstruction, epidural anesthesia, prophylactic corticosteroid use, and duration of anesthesia. The thoracic approach was either an open or minimally invasive (thoracoscopic, mediastinoscopy-assisted, or robot-assisted) esophagectomy; however, information on the thoracic approach was not recorded before April 2014. Additionally, the database did not record whether the abdominal approach was an open laparotomy or laparoscopy. Lymph node dissection was either a 2-field (thoracic and abdominal) or 3-field (cervical, thoracic, and abdominal, with or without supraclavicular lymph node dissection) approach. In Japan, 3-field lymph node dissection generally involves a cervical anastomosis, whereas 2-field lymph node dissection involves an intrathoracic anastomosis.^6^ Prophylactic corticosteroid use was defined as the administration of intravenous methylprednisolone or hydrocortisone on the day of surgery.^7–9^ Duration of anesthesia was categorized into quartiles.

Additionally, we examined hospital background factors (i.e., type of hospital and hospital volume) and fiscal year. The type of hospital was either teaching or non-teaching; hospital volume was defined as the number of esophagectomies performed annually in each hospital, and categorized into quartiles.

References

[1] Quan H, Sundararajan V, Halfon P, et al. Coding algorithms for defining comorbidities in ICD-9-CM and ICD-10 administrative data. Med Care. 2005;43(11):1130–1139. https://doi.org/10.1097/01.mlr.0000182534.19832.83.

[2] Quan H, Li B, Couris CM, et al. Updating and validating the Charlson comorbidity index and score for risk adjustment in hospital discharge abstracts using data from 6 countries. Am J Epidemiol. 2011;173(6):676–682. https://doi.org/10.1093/aje/kwq433.

[3] Bannay A, Chaignot C, Blotière PO, et al. The best use of the Charlson comorbidity index with an electronic health care database to predict mortality. Med Care. 2016;54(2):188–194. https://doi.org/10.1097/MLR.0000000000000471.

[4] Hirano Y, Kaneko H, Konishi T, et al. Impact of body mass index on major complications, multiple complications, in-hospital mortality, and failure to rescue after esophagectomy for esophageal cancer: a nationwide inpatient database study in Japan. Ann Surg. 2023;277(4):e785-e792. https://doi.org/10.1097/SLA.0000000000005321.

[5] WHO Expert Consultation. Appropriate body-mass index for Asian populations and its implications for policy and intervention strategies. Lancet. 2004;363(9403):157–163. https://doi.org/10.1016/S0140-6736(03)15268-3.

[6] Watanabe M, Toh Y, Ishihara R, et al. Comprehensive registry of esophageal cancer in Japan, 2014. Esophagus. 2022;19(1):1–26. https://doi.org/10.1007/s10388-021-00879-1.

[7] Hirano Y, Konishi T, Kaneko H, et al. Impact of prophylactic corticosteroid use on in-hospital mortality and respiratory failure after esophagectomy for esophageal cancer: Nationwide inpatient data study in Japan. Ann Surg. 2023;277(6):e1247-e1253. https://doi.org/10.1097/SLA.0000000000005502.

[8] Kuwano H, Nishimura Y, Oyama T, et al. Guidelines for diagnosis and treatment of carcinoma of the esophagus April 2012 edited by the Japan Esophageal Society. Esophagus. 2015;12(1):1–30. https://doi.org/10.1007/s10388-014-0465-1.

[9] Kitagawa Y, Uno Y, Oyama T, et al. Esophageal cancer practice guidelines 2017 edited by the Japan esophageal society: part 2. Esophagus. 2019;16(1):25–43. https://doi.org/10.1007/s10388-018-0642-8.

**Supplemental Table 1. ICD-10 and procedure codes used to identify postoperative complications**

|  | **ICD-10 codes** | **Procedure codes as original Japanese codes** |
| --- | --- | --- |
| Respiratory complications | J12–J18, J80, J96, J690, J691, J958, J959 | Mechanical ventilation use lasting >2 days following surgery |
| Anastomotic leakage | T813 | Long-term drainage tube placement (defined as placement for ≥3 weeks after surgery) and procedures for anastomotic leakage |
| Pneumothorax | J93 | Procedures for pneumothorax |
| Chylothorax | I898, S278, T812 | Procedures for chylothorax |
| Empyema | J860, J869 | Procedures for empyema |
| Peritonitis | K65 | Procedures for peritonitis |
| Ileus/bowel obstruction/symptomatic hernia (hiatal or diaphragmatic) | K440, K441, K449, K560, K562, K565–K567, K913 | Procedures for ileus/bowel obstruction or hiatal/diaphragmatic hernia |
| Pulmonary embolism | I26 | None |
| Acute coronary syndrome | I21–I25 | None |
| Heart failure | I50 | None |
| Stroke | I60–I66 | None |
| Acute kidney injury | N17 | None |
| Sepsis | A021, A227, A241, A267, A282, A327, A394, A40, A41, A548, B007, B349, B377, P36 | None |

ICD-10, International Classification of Diseases, Tenth Revision.

**Supplemental Table 2. Subcategories of other major complications categorized by hospitals’ early extubation proportion**

|  | **Very low (<11%)**  **(*n*=9407)** | **Low (11–37%)**  **(*n*=9395)** | **Medium (38–83%)**  **(*n*=9621)** | **High (≥84%)**  **(*n*=9560)** | ***P* value** |
| --- | --- | --- | --- | --- | --- |
| Pneumothorax | 165 (1.8) | 198 (2.1) | 130 (1.4) | 110 (1.2) | <.001 |
| Chylothorax | 102 (1.1) | 123 (1.3) | 115 (1.2) | 113 (1.2) | .57 |
| Empyema | 104 (1.1) | 120 (1.3) | 131 (1.4) | 132 (1.4) | .32 |
| Peritonitis | 118 (1.3) | 110 (1.2) | 109 (1.1) | 101 (1.1) | .64 |
| Ileus/bowel obstruction/symptomatic hernia | 131 (1.4) | 149 (1.6) | 162 (1.7) | 84 (0.9) | <.001 |
| Pulmonary embolism | 47 (0.5) | 29 (0.3) | 35 (0.4) | 26 (0.3) | .049 |
| Acute coronary syndrome | 20 (0.2) | 15 (0.2) | 29 (0.3) | 23 (0.2) | .23 |
| Heart failure | 162 (1.7) | 232 (2.5) | 173 (1.8) | 153 (1.6) | <.001 |
| Stroke | 51 (0.5) | 37 (0.4) | 42 (0.4) | 24 (0.3) | .016 |
| Acute kidney injury | 76 (0.8) | 75 (0.8) | 55 (0.6) | 41 (0.4) | .001 |
| Sepsis | 192 (2.0) | 188 (2.0) | 160 (1.7) | 144 (1.5) | .012 |
| Others resulting in death | 4 (0.0) | 20 (0.2) | 15 (0.2) | 5 (0.1) | .001 |

Data are presented as *n* (%).
